# Flagellin, a plant-defense-activating protein identified from *Xanthomonas axonopodis* pv. *Dieffenbachiae* invokes defense response in tobacco

**DOI:** 10.1186/s12866-023-03028-z

**Published:** 2023-10-05

**Authors:** Tamilarasi Mani, J. Beslin Joshi, R. Priyadharshini, Jeya Sundara Sharmila, Sivakumar Uthandi

**Affiliations:** 1 Biocatalysts Laboratory, Department of Agricultural Microbiology, Directorate of Natural Resource Management, Tamil Nadu Agricultural University, Coimbatore, 641 003 India; 2 Present Address: Centre for Water Resources Development and Management, Kozhikode, India; 3 Department of Nano Science and Technology, Directorate of Natural Resource Management, Tamil Nadu Agricultural University, Coimbatore, India; 4 Present Address: Department of Microbiology, Karpagam Academy of Higher Education, Coimbatore, India

**Keywords:** Secretome, *Xanthomonas axonopodis*, Flagellin, Plant-microbe interaction, Defense-related enzymes, Defense genes

## Abstract

**Background:**

Secretome analysis is a valuable tool to study host-pathogen protein interactions and to identify new proteins that are important for plant health. Microbial signatures elicit defense responses in plants, and by that, the plant immune system gets triggered prior to pathogen infection. Functional properties of secretory proteins from *Xanthomonas axonopodis* pv. *dieffenbachiae* (Xad1) involved in priming plant immunity was evaluated.

**Results:**

In this study, the secretome of Xad1 was analyzed under host plant extract-induced conditions, and mass spectroscopic analysis of differentially expressed protein was identified as plant-defense-activating protein viz., flagellin C (FliC). The flagellin and Flg22 peptides both elicited hypersensitive reaction (HR) in non-host tobacco, activated reactive oxygen species (ROS) scavenging enzymes, and increased pathogenesis-related (PR) gene expression viz., *NPR*1, *PR1*, and down-regulation of *PR2* (β-1,3-glucanase). Protein docking studies revealed the Flg22 epitope of Xad1, a 22 amino acid peptide region in FliC that recognizes plant receptor FLS2 to initiate downstream defense signaling.

**Conclusion:**

The flagellin or the Flg22 peptide from Xad1 was efficient in eliciting an HR in tobacco *via* salicylic acid (SA)-mediated defense signaling that subsequently triggers systemic immune response epigenetically. The insights from this study can be used for the development of bio-based products (small PAMPs) for plant immunity and health.

**Supplementary Information:**

The online version contains supplementary material available at 10.1186/s12866-023-03028-z.

## Background

Several biotic and abiotic factors threaten plants during their life span, and about 16% of global yield loss is attributed to plant diseases [1]. Plant pathogens are of significant concern because of the associated primary and secondary yield loss, thereby affecting the food quantity and quality, resulting in huge losses. Among the different plant pathogenic genera, *Xanthomonas* was of great importance ascribed to the potential damage caused in economically important crops like rice, citrus, cassava, tomato, beans, passion fruit, sugarcane, and banana [2]. Their ability to survive in seed, soil, insects, weeds, and non-host plants favored the epidemics [3]. Despite several approaches including the use of resistant varieties, windbreaks, disinfestation of farm implements, the addition of copper-based chemicals, contaminated plants eradication, etc., for pathogen management [4, 5], it has been insignificant on the grounds of environmental pollution, copper toxicity and the emergence of new virulent races [6]. Consequently, considerable attention has been paid to the development of bioactive products from various sources.

In general, *Xanthomonas* genus deploys several sets of strategies to interact with the host, and the successful interaction depends on the protein secretory systems that secrete proteins into the extracellular milieu or transport proteins directly into the cytosol of the host cell. *Xanthomonas* encode six types of protein secretion systems. Type one secretion system has the simplest structure and allows direct secretion of bacterial effectors from the cytosol to the outer environment. While Type two, and five secretion systems transport proteins across the inner membrane *via.*, sec, or TAT pathway; Type three, four, and six secretion systems are associated with extracellular pilus structure and translocate bacterial proteins directly into the host cell. The Type two secretion system delivers apoplastic effectors and plant cell wall-degrading enzymes, whereas the Type three secretion system (T3SS), delivers effector proteins into the cytoplasm of plant cells [7]. Effector proteins act to suppress or manipulate host processes by targeting a variety of cellular process, and defense components, such as receptor kinases, signaling pathways, programmed cell death, ubiquitination, vesicle trafficking, and cell wall reinforcement. Bacteria have developed strategies to invade plants using plant-like proteins, including E3 ligases, F-box proteins, etc., to increase pathogenicity [8]. Plants have evolved to recognize these effectors, directly or indirectly, *via*., the products of resistance genes. Plants have receptor-like kinases (RLK) and receptor-like proteins (RLP) as immune receptors in the plasma membrane to recognize the pathogen attack *via*., pathogen-associated molecular signatures or patterns (PAMPs), which are conserved across microbial classes and essential for pathogen survival. The recognition of different PAMPs by specific pattern recognition receptors initiates PAMP-triggered immunity (PTI). Recognition of pathogen-associated molecular signatures is characterized by defense cascade reactions in resistant plants and induces HR. Resistance development relies on the activation of mitogen-activated protein kinases (MAPK), production of reactive oxygen species (ROS), transcriptional re-programming, hormone biosynthesis, and callose deposition that ultimately leads to localized cell death and has a major role in plant basal immunity [9]. Microbial secretome analyses are useful for the identification of secreted proteins involved in host plant manipulation by regulating symbiosis and quorum sensing [10]. Besides understanding plant-microbe interaction, protein secretion studies will provide new insight into handling microbes for sustained and improved plant health. Flagellin from plant pathogenic bacteria is a well-known pathogen-associated molecular signature that elicits the first line of defense in plants.

Perception of the conserved peptides within the flagellar proteins by pattern recognition receptors in plants transduces a series of signaling events through MAPK cascade and WRKY transcription factors to confer enhanced resistance. The flagellar peptides (15 to 22 amino acid length) manifested a stronger defense response compared to whole flagellar protein and different amino acid sequences in plant-associated bacteria *viz., Rhizobium* and *Agrobacterium* failed to induce defense reaction [11, 12]. Similarly, we demonstrated that recombinantly over-expressed flg22 peptide of *Rhizobium* sp. exerted HR on the non-host plants (unpublished data, Suraj et al., 2020). Hitherto, in our previous study, the *Xanthomonas axonopodis* pv. *punicae* hpaG effector mediated defense response in the non-host plant tobacco was investigated (unpublished data), and those results suggest that plant-defense-activating proteins from plant pathogenic bacteria can be effectively utilized for plant immunity. With this perspective, we studied the secretome of plant pathogenic bacterium, *Xanthomonas axonopodis* pv. *dieffenbachiae* and identified plant-defense-activating protein (flagellin), efficient in triggering HR, thereby activating defense response genes in non-host tobacco.

## Results

### Discerning the protein harvest phase in Xad1 and secretory protein production from Xad1

**Fig. 1:**
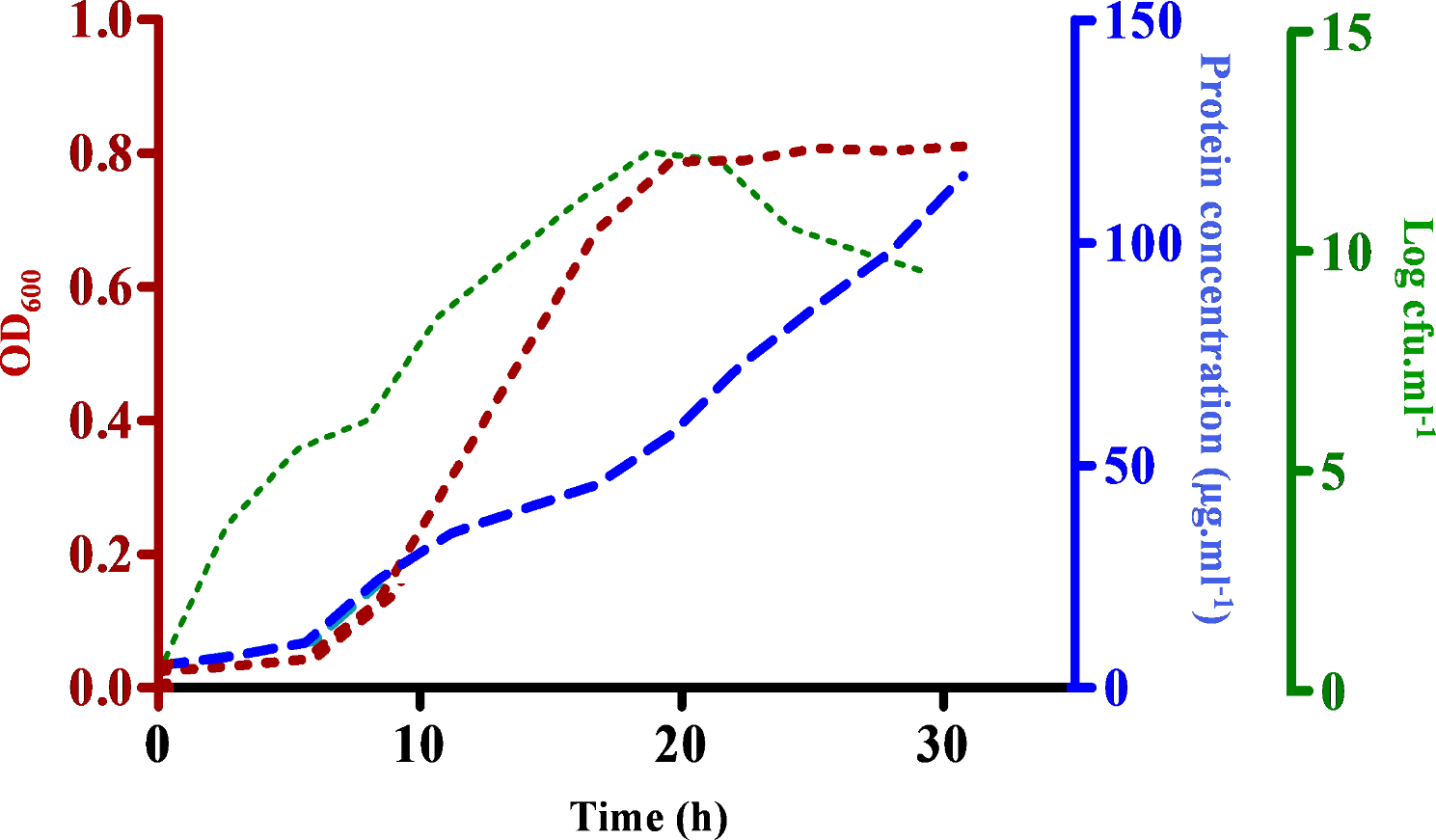
Growth curve and protein production of *Xanthomonas axonopodis *pv. *dieffenbachiae* in M9 broth. Blue line indicates OD at 600 nm, red line indicates protein concentration (μg.mL^− 1^), and green line indicates Log of cfu.mL^− 1^

Growth and protein secretion of *Xad*1 in M9 minimal broth were monitored for 36 h by recording the OD, colony-forming units (CFU), and protein concentration at regular intervals. From an initial lag phase of 6 h, the cells entered into the log phase and continued their growth steadily up to 18 h. The active decline phase began after 21 h with a sharp decrease in CFU. Cell death occurs after the transition from logarithmic to stationary phase as indicated by the drop of CFU at 18 h from 146 × 10^12^ cfu.mL^− 1^ to 90 × 10^11^ cfu.mL^− 1^ at 21 h (Fig. 1). Differential expression of *Xad*1 secretome was studied by growing *Xad*1 cells in M9 medium supplemented with *Dieffenbachiae* leaf extract. The secreted protein from the culture supernatant of *X. axonopodis* pv. *dieffenbachiae* was concentrated by ammonium sulfate precipitation, followed by desalting and clarification. Separation of the secreted protein fraction by 1D SDS-PAGE revealed an additional band under plant extract-induced conditions compared to uninduced culture (Fig. S1A). In this study, 1D SDS-PAGE analysis of *Xad*1-induced protein fraction showed the differentially expressed protein around 29 kDa, and 2D SDS-PAGE revealed three isoforms at the corresponding molecular weight of 29 kDa (Fig. S1B).

### Differentially expressed protein was identified as flagellin, FliC

The differentially expressed protein in the *Xad*1 secretome was identified by MALDI-TOF analysis. The *m/z* values and the peak file obtained were analyzed in the MASCOT server (Fig. S1C). Our results from peptide mass fingerprinting analysis indicated FliC or flagellin protein as a top matching hit with a score of 1319.94 possessing nine unique peptide matches and a query coverage of 39.94. The amino acid sequence from *Xad*1 secretome had a high level of similarity to flagellar proteins (FliC) of *E. vulneris* NBRC 102,420; which is similar to *X. campestris* pv. *citri* FliC sequence. Parallel to these results, the MASCOT analysis performed using the mass-charge (*m/z*) value obtained from Sandor Proteomics matches with flagellar protein and flagellin-related hook proteins.

### Xanthomonas flagellin communicates via FLS2 in plants

**Fig. 2:**
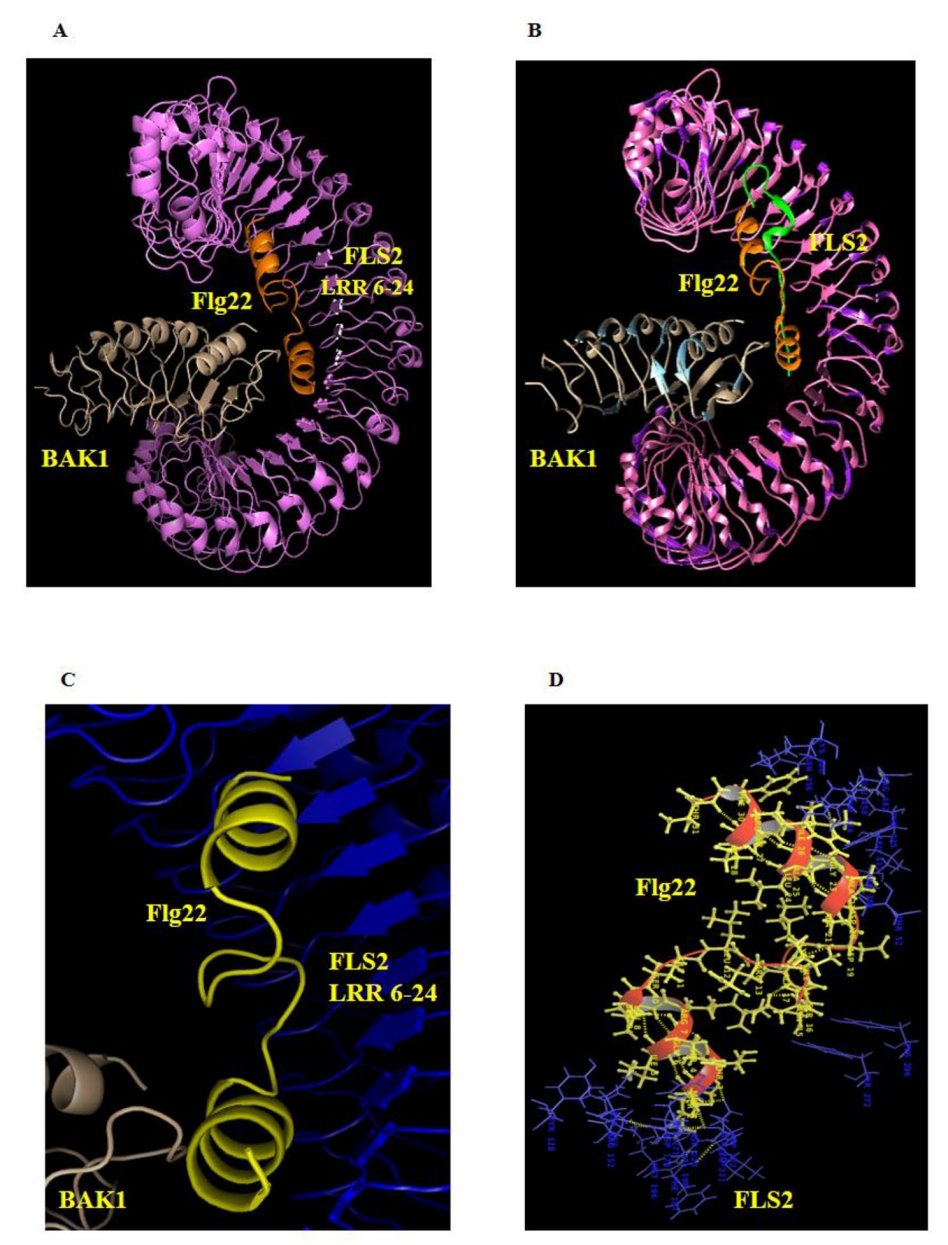
Molecular docking analysis of *Xad*1_Flg22_(orange) with FLS2 (violet) and BAK1 (wheat) complex. (**A**) The Flg22_Xad1_ peptide regions, when docked with the FLS2-BAK1 complex, revealed Flg22_Xad1_ binds to a shallow groove at the inner surface of the FLS2-LRR as found in the crystal structure of the solved Flg22-FLS2-BAK1 complex (PDB: 4MN8). (**B**) The superimposition of the docked complex (Flg22_Xad1_-FLS2-BAK1 in orange-violet-wheat) with the crystal structure 4MN8 (Flg22-FLS2-BAK1 in green-pink-blue) had similar folding and positioning in the structure alignment (**C**) The Flg22_Xad1_ (yellow) interacted with the FLS2 (blue) spanning LRR 6–17. (**D**) The interaction of Flg22_Xad1_(yellow) with FLS2 (blue) was stabilized through H-bonding (yellow dash) and hydrophobic interaction

The FliC protein sequence of *Xad*1 showed similarity with many flagellin homolog structures, including 3k8v, 3a5x, 4nx9, etc. FliC_Xad1_ and 1UCU shared 43.6% identity, and the primary amino acid sequence was used for 3D structure prediction using 1UCU as a template (Fig. 2A). The model was validated in the SAVES server. The Ramachandran plot revealed that more than 98% of the amino acids were present in the allowed region. The flagellin needs to be disassociated to make D0–D1 accessible to innate immune receptors [13]. *In planta*, flagellin is sensed by the flagellin sensitive 2 (FLS2) receptor *via* the interaction of 22–31 amino acids in the N-terminal region of FliC protein (Fig. S2A, B). Sequence homology of Xad1_Flg22_ peptide: epitope region with other important bacterial strains is depicted in supplementary Figure S3.

Homology modeling of the flagellin epitope region spanning 33 amino acids (N-terminalASMSTSIQRLSSGLRINSAKDDAAGLAISERC-terminal) with 77% sequence identity to PDB: 1UCU was used as a template for docking analysis with the FLS2-BAK1 complex. Protein-protein docking for the predicted Flg22_Xad1_ structures was used as ligand, and the FLS2 receptor of *Arabidopsis thaliana* along with the BAK complex (PDB: 4MN8) as a receptor and the interaction is presented in Fig. 2A.The docking analysis revealed Flg22_Xad1_interaction with FLS2 receptor was similar to the crystal structure of the FLS2LRR-flg22-BAK1LRRcomplex. The superimposition of the docked complex with FLS2LRR-flg22-BAK1LRR (4MN8) showed no significant deviation in the alignment of alpha-carbon atoms and root mean square deviation of the structural alignment, which was 1.6Å (Fig. 2B). The Flg22 formed H-bonding and hydrophobic contact with the LRR6-24 of the FLS2 (Fig. 2C, D).

### Flagellin elicits a defense response in the non-host plant

**Fig. 3:**
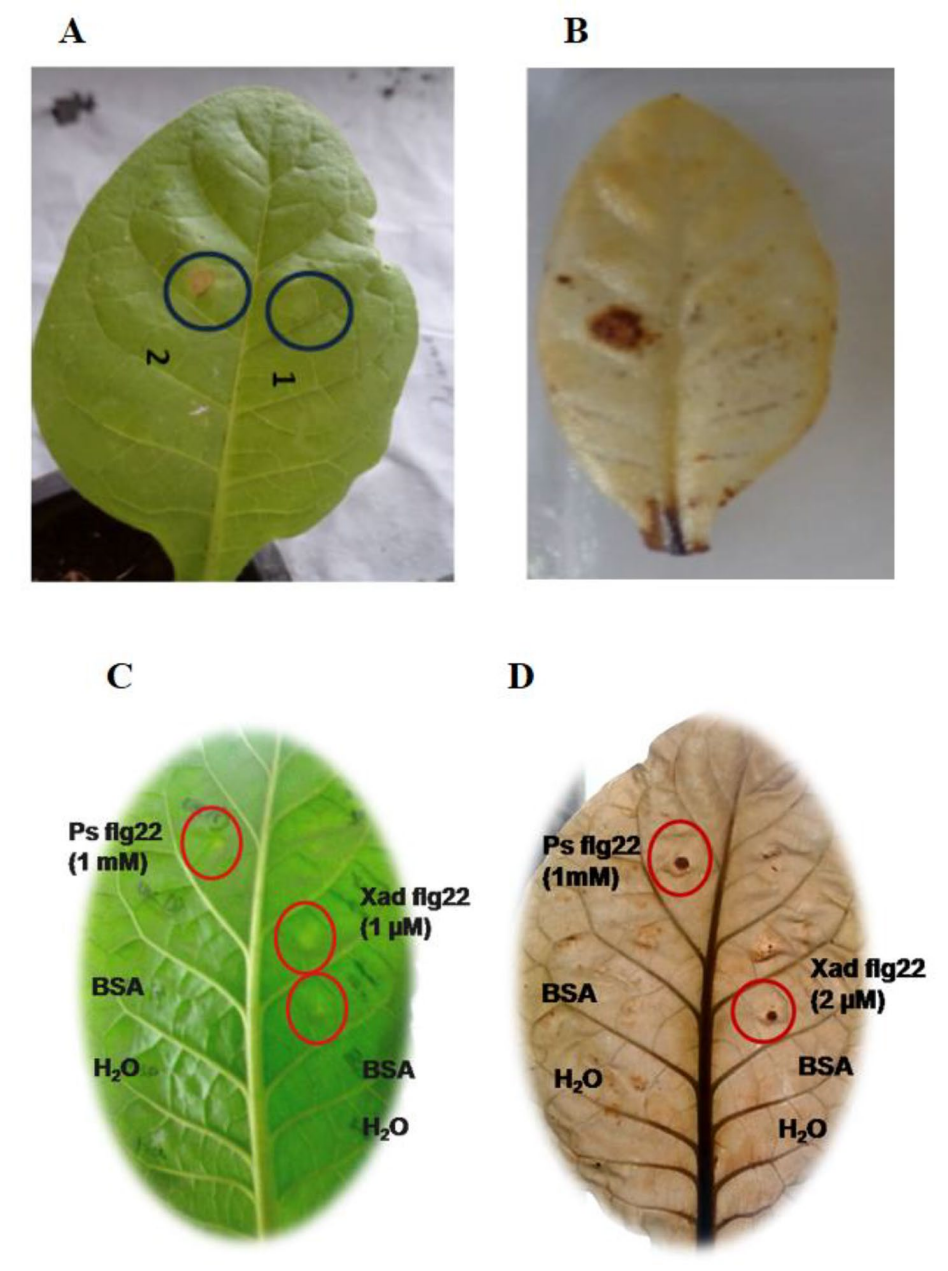
Hypersensitive response and hydrogen peroxide detection in tobacco leaves: upon flagellin elicitation (**A**, **B**) and upon Flg22_Xad1_ elicitation (**C**, **D**)

In the present study, infiltration of the concentrated secretome of *Xad*1 containing flagellin induced defense response in tobacco plants within 24 h, followed by necrosis at the infiltrated spot (Fig. 3A). Further to confirm the defense elicitation specific to flagellin protein of *Xad*1, the 22-N-terminal amino acid sequence determined by MALDI-TOF analysis was synthesized and evaluated for defense response. Similar to *Xad*1 flagellin protein, the Flg22_Xad1_ peptide also initiated a similar incompatible reaction (HR) in tobacco plants similar to positive control, Flg22(*Ps*) (Fig. 3C). Our results suggest that the flagellin (FliC) and its peptide (Flg22) were both capable of eliciting a defense response in non-host tobacco.

### Flagellin activates pathogenesis-related process in plant system

In the present study, the initiation of defense response by ROS burst in response to flagellin sensing in tobacco plants was detected by hydrogen peroxide accumulation at the site of infiltration (in both flagellin and Flg22; Fig. 3B, D). Hydrogen peroxide accumulation at Flg22 infiltrated site was observed as dark brownish-black precipitate compared to the water infiltrated spot, suggesting the flagellin epitope region (Flg22) initiated a stronger defense response at a lower concentration (2 μM) compared to FliC protein (Fig. 3B, D).

**Fig. 4:**
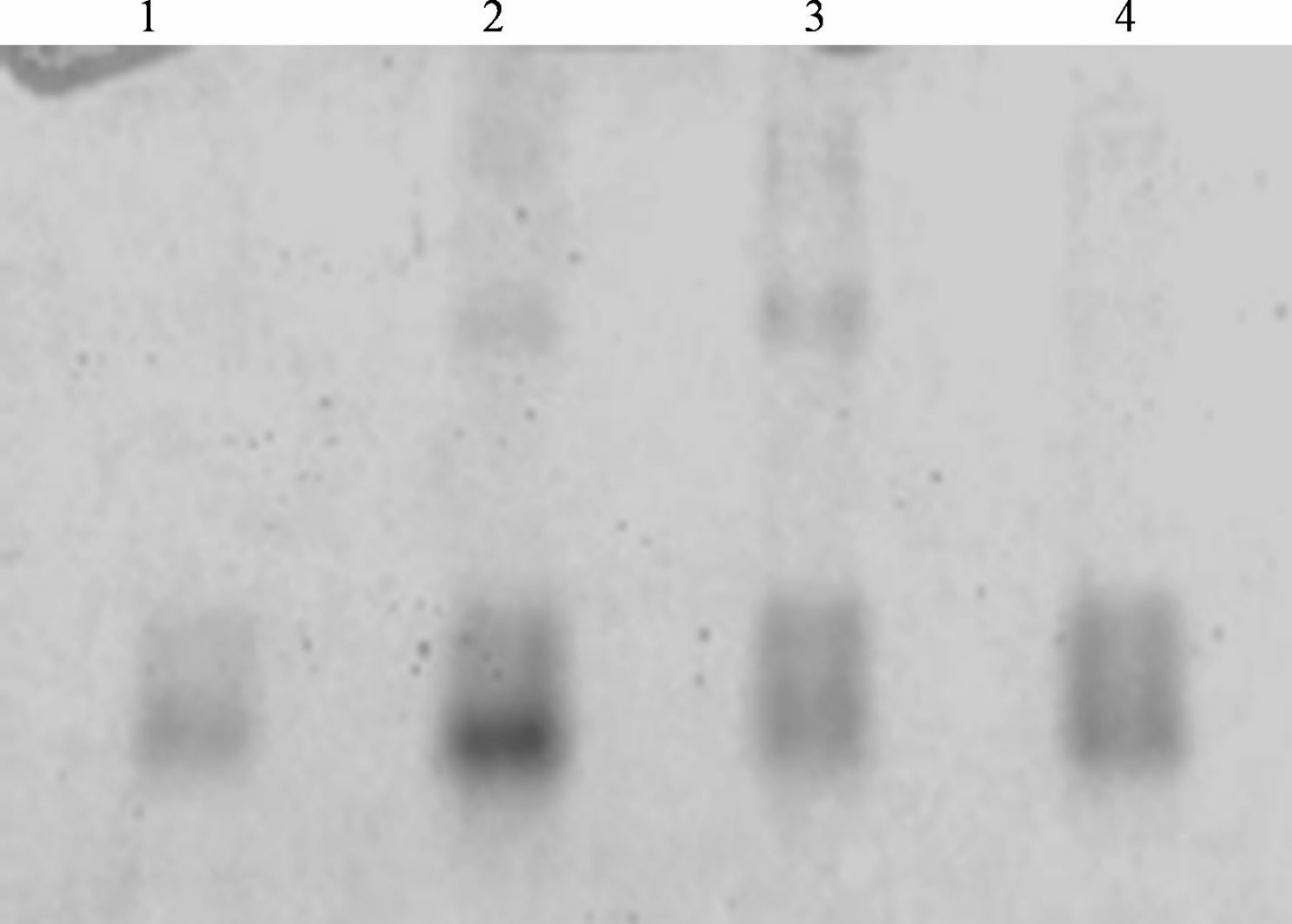
Native PAGE for PPO activity under non-denaturing conditions. Polyphenol oxidase (PPO) bands revealed by staining with 10 mM catechol in phosphate buffer. Lane 1 Buffer; Lane 2 FliC_Xad1_; Lane 3 Heat killed protein; Lane 4 Control

**Fig. 5:**
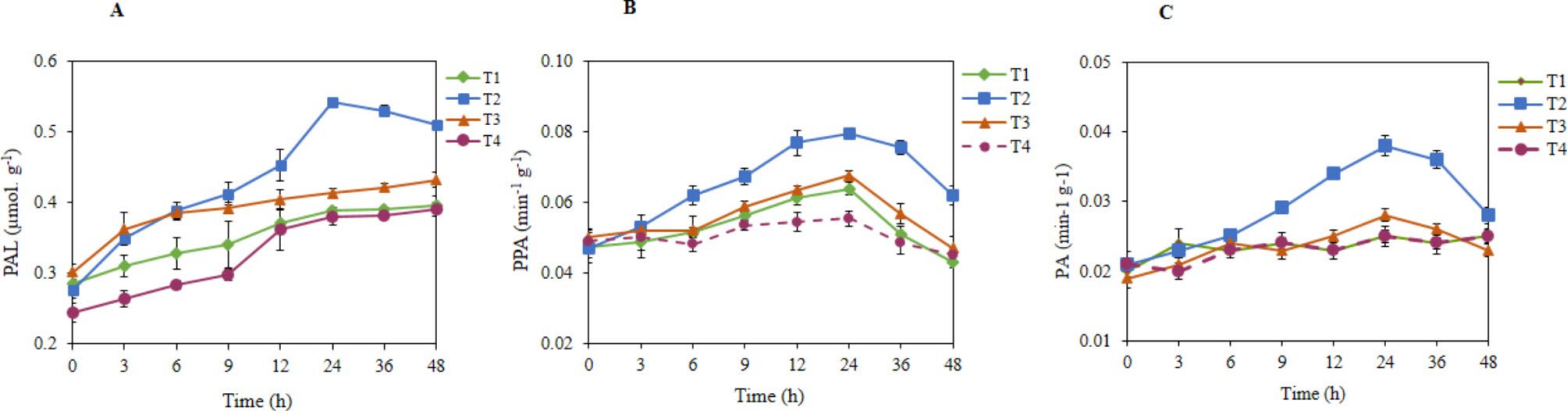
FliC_Xad1_ induced defense-related enzyme assay in tobacco. Polyphenoloxidase activity (PPA) (**A**); Peroxidase activity (PA) (**B**); Phenylalanine ammonia-lyase activity (PAL) (**C**).T_1_, Buffer; T_2_, FliC_Xad1_; T_3_, Heat killed protein; T_4_, Untreated control

**Fig. 6:**
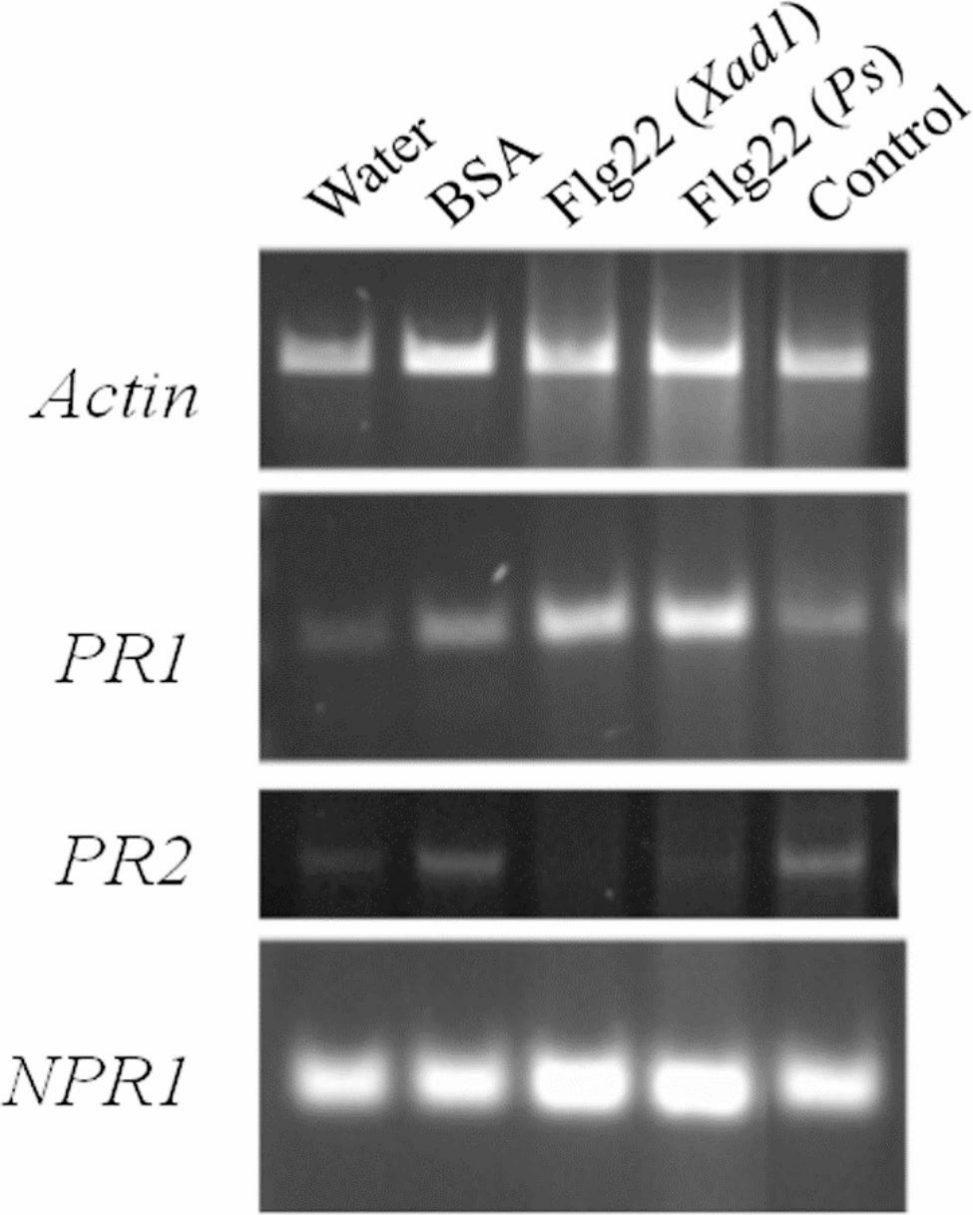
Differential expression of *PR* gene in response to Flg22_Xad1_. Pathogenesis-related genes *PR1*, *PR2*, and *NPR1* were amplified using specific primers from the tobacco leaves infiltrated after 24 h with water, BSA, Flg22(*Xad1*), Flg22(*Ps*), and control (without infiltration). The transcript levels across the samples were normalized using the housekeeping gene, *Actin*

Formation of H_2_O_2_ upon elicitation was one of the earliest events, critical for the activation of hypersensitive cell death. Several scavenging enzymes ameliorated cell toxicity due to ROS bursts. Analyses of ROS scavenging enzymes revealed the early onset of their activity in the infiltrated leaves. The PPO activity increased from the 3rd h of post-flagellin treatment, and it reached the maximum (0.12 min^− 1^.g^− 1^ of leaf tissue) at 24 h after elicitation with different PPO isoforms suggesting that the flagellin treatment might have upregulated three PPO isoforms (Fig. 4 A). However, there was no discernible difference in either isoform number or their intensity between the buffer and plant control (Fig. 4). Peroxidase activity was found to be more in flagellin treatment and was found maximum (0.038 U.mg^− 1^ of leaf tissue) during the 24th h post-treatment (Fig. 5B). Flagellin elicitation resulted in the increased activity of phenylalanine ammonia-lyase from 3rd h of post-treatment and reached to a maximum of 0.379 μmol of cinnamic acid in ^− 1^.g^− 1^ during the 24th h post-treatment (Fig. 5C). Then it gradually decreased as recorded. To verify whether Flg22_Xad1_ elicitation induces plant systemic resistance, the expression of non-expresser of PR genes 1 (*NPR1*), pathogenesis-related gene 1 (*PR1*), and pathogenesis- related gene 2 (*PR2*) were studied. The expression of *NPR1* and *PR1* genes were found to be up-regulated by Flg22(*Xad1*) and Flg22(*Ps*) elicitor, while *PR2* was down-regulated or silenced (Fig. 6).

## Discussion

*X. axonopodis* pv. *dieffenbachiae* is a biotrophic Gram-negative gamma proteobacteria that causes blight disease in ornamental plants. It has been reported that most of the *Xad*1 cells in the culture were viable during the lag, and log phases, and was indicated by similar curve progression of OD and CFU from 0 to 26 h. The extracellular protein concentration showed a gradual linear rise with increasing cell density and low CFU during the period suggesting cell lysis might account for the increased protein levels as reported previously [14]. The depletion of phosphorous, glucose, oxygen, and nitrogen during the growth phase might influence protein synthesis and its composition. The problem encountered by increasing levels of cytosolic protein due to the lysis of cells into the extracellular space can be carefully avoided by harvesting the cells during the early stationary phase.

Bonas (1994) [15] has established that Xanthomonads interactions with the host are under the control of *hrp* genes. Many *hrp* genes encode proteins that are components of the hrp secretion pathway and other *hrp* genes in the regulation of *hrp* and *avr* genes [16]. The *hrp* genes of bacteria are induced only during their interaction with the host plant. The expression of *hrp* genes can also be induced in vitro when bacteria are grown in a defined minimal medium of low pH and by the addition of certain sugars or sugar alcohols as carbon sources [17]. M9 minimal medium mimics the conditions the pathogen encounters in the apoplast [18, 19]. Rico and Preston (2008) [20] showed that the addition of apoplast extracts to M9 minimal medium promoted a higher level of *hrp* gene expression than synthetic minimal media. In the present study, to induce the proteins involved in host-pathogen interaction, the *Xad*1 was grown in M9 minimal medium with host plant extract as it mimics the plant apoplastic fluid. In our study, approximately 1-1.5 mg.mL^− 1^ of protein was obtained from 200 mL of the medium. Similarly, the addition of tuber extract induced approximately 50–60 proteins, of which 35 proteins were related to known virulence factors, such as pectic enzymes, protease, and the avirulence protein compared to the un-induced condition [21]. Watt et al. (2009) [14] reported that plant molecules play an essential role in triggering the bacterium to activate its protein secretion system, and protein appearing under induced conditions did not show any similarity with plant protein. Although the trigger molecules in the plant extract remain unknown, their biological properties must be conserved, so that bacterial cells endure active to secrete pathogenic factors into the culture medium [22].

Flagellin is the main proteinaceous component of extracellular flagellum filaments and is essential for the mobility and ability of bacteria to infect host plants. Similar to our results, the 1D SDS-PAGE detected the flagellin core protein (FliC) from the proteins of outer membrane vesicles by growing *X. campestris* pv. *campestris* (*Xcc*) in M9 medium. Besides flagellin, a few intracellular proteins, cellulase Cel5, and proteases (PrtA, PrtB, PrtC) were detected from the *Erwinia chrysanthemi* wild-type strain 3937 secretome [23]. Flagellar proteins were found as a significant component in the extracellular proteome of Gram-negative bacteria [24]. Multiple isoforms of FliC with molecular mass from 20 to 29 kDa and p*I* values from 3.5 to 4.5 were identified. The release of flagellar proteins into the extracellular medium is commonly observed in bacterial cultures [25]. The flagellum is easily disrupted from the cell surface, particularly under shaking cultures, and is not excluded by 0.2 μm filters. Komoriya et al., (1999) [25] detected 12 spots in 2D electrophoresis of 29 kDa flagellin protein, and the different isoforms might have arisen due to glycosylation and natural degradation by extracellular proteases. Similarly, our analysis of *Xad*1-induced protein fraction showed the differentially expressed protein around 29 kDa, and 2D SDS-PAGE revealed three isoforms at the corresponding molecular weight of 29 kDa.

The FliC consists of four domains designated D0, D1, D2, and D3. D0 to D1 was well conserved across all bacterial flagellins, and D2 to D3 domains are highly variable. Hydrophobic residues rich, N- and C-termini form coiled-coil interfaces that enable them to associate with one another and filament polymerization. The flagellum has three regions viz., basal body, hook, and filament, and the filament is composed of about 20,000 flagellins (FliC) proteins that are incorporated below the distal pentameric FliD cap [26]. In *Arabidopsis*, perception of the bacterial protein, flagellin, occurs at the plasma membrane through the FLS2 and BRI1-Associated Kinase1 (BAK1) complex. FLS2 binds to Flg22, a 22-amino acid flagellin-derived peptide that is a pathogen-associated molecular pattern (PAMP). Once bound to Flg22, the complex initiates several defense responses [27]. The FLS2 has an extracellular domain with 28 LRRs from the interaction site for flagellin. Binding sites with high-affinity and specificity for Flg22 have been biochemically characterized in tomato (*Lycopersicon esculentum*), and *Arabidopsis* [28, 29], and a single point mutation in one of the LRR repeats affects the binding activity of Flg22 completely. The crystal structural studies of FLS2 ectodomains and BAK1 in complex with Flg22 showed the mechanisms underlying Flg22 perception by FLS2. Flg22 binds to the concave surface of the FLS2 superhelical ectodomain spanning 14 LRRs (LRR3-16) [30]. The Flg22-bound FLS2 ectodomain directly interacts with the BAK1 ectodomain, with the C-terminal region of the FLS2-bound Flg22 stabilizing the FLS2-BAK1 dimerization by acting as ‘‘molecular glue’’ between the two ectodomains. Thus, the FLS2-BAK1 heterodimerization was both ligand and receptor-mediated [31, 32]. FLS2 has been identified in tomato, tobacco, rice, *Brassica*, poplar, and grapes, and their wide distribution across plant species suggests FLS2-mediated flagellin perception was evolutionary [33].

Arlat et al. (1992) [34] found that the concentrated supernatant preparations of the plant pathogenic bacterium *Pseudomonas solanacearum* induced the same pattern of response on *Petunia* sp. Recognition of pathogen infection in plants provokes HR-based programmed cell death (PCD) to limit the further spread of the pathogen by killing the pathogen and infected host cells [35].

The HR response of Flg22_Xad1_ from *X. axonopodis* pv. *dieffenbachia* was analogous to Flg22 from *Pseudomonas syringae*. Flg22 from *X.campestris* pv. *campestris, X. citri* ssp. *citri* and *Xanthomonas axonopodis* pv. *citri* was reported to elicit defense response in *Arabidopsis* [30], citrus [36], and tomato [37], respectively. Though flagellin had been identified from both beneficial and pathogenic bacteria, the Flg22 peptide activity had been widely studied from the genera *Pseudomonas* and *Xanthomonas* [38]. The five amino acids viz., Arg_294_, Tyr_296_, Gly_318_, Ser_320_, Asp_414_ determine the flagellin recognition and single amino acid polymorphism in Flg22 peptide or epitope binding region modulates the defense response [30]. Plant pathogenic bacteria avoid host detection of their flagella by expressing non-recognized flagellins [39–41]. Even within a single pathogen taxon (a single species or pathovar), different strains can express different flagellins and have varied host elicitation activities. For instance, in *Xanthomonas campestris* pv *campestris* (*Xcc*) strains, the defense-eliciting activity of flagellins was determined by flg22 region carrying Val-43/Asp polymorphism and its interaction with *Arabidopsis thaliana* FLAGELLIN SENSING2 (FLS2), a transmembrane leucine-rich repeat kinase, confers flagellin responsiveness. *Arabidopsis* FLS2 detected flagellins carrying Asp-43 or Asn-43 but not Val-43 or Ala-43, and it responded minimally for Glu-43 [28], thereby determining the eliciting/non-eliciting nature of *Xcc* flagellins. Similarly, a mutation in *Xanthomonas oryzae* pv. *oryzae* flagellin failed to elicit the HR response in rice cells [33]. Flagellin glycosylation or post-translational modification determines their interaction with receptors and hypersensitive inducing response [42]. Glycosylation of flagellin was reported to determine the compatible and incompatible reactions in plants. Glycosylation may increase the hydrophobicity of flagella which might interfere with efficient recognition by host plants.Flg22 and similar peptides with conserved N-terminal domain of flagellins from *Pseudomonas aeruginosa* and other bacteria elicit plant defense responses but do not activate HR in non-host plants while flagellins from *P. avenae*, *P*. *syringae* pv. glycinea and *P*. *syringae* pv. tomato-induced cell death [11]. The immune elicitation potential between flagellin monomers and their peptide varies [11, 43–46].

During the pathogen attack, a burst in reactive oxygen species (ROS), namely singlet oxygen, superoxide anion, hydrogen peroxide, and hydroxyl radicals, occurs at the site of infection. ROS plays a central role in signaling and initiating a cascade of events related to plant innate immunity [47]. Among the ROS, hydrogen peroxide is the most stable and can be fixed in acidic 3,3-diaminobenzidine (DAB) [46–48]. Flg22 peptide was reported to elicit HR at a concentration of 10 to 100 μM concentration and, in some cases, even at a very minimum concentration of 0.1 μM [49, 50]. It was reported that Flg22 from *Xanthomonas* sp induced defense response at a minimum concentration of 0.1 to 0.2 μM concentration in tomato and citrus [36, 37]. In 2014, Mott and his co-workers suggested the impact of the elicitor’s nature (larger protein or smaller peptide), their apoplastic concentrations on its stability, apoplast motility, and receptors interaction to induce defense response [46].

The highest PPO activity might be the resultant effect of protein on the plant immune system [51]. Increased peroxidase (PO) has been observed in several resistant interactions involving plant pathogenic fungi, bacteria, and viruses [52]. The PO activities are linked to lignification and generation of hydrogen peroxides at later stages of infection, which inhibit the pathogens directly, or the generation of other free radicals with antimicrobial effects [53, 54] that restrict the development of challenging phytopathogenic bacteria [55]. Phenylalanine ammonia-lyase plays a vital role in the biosynthesis of phenolic phytoalexins [56], and during the initial establishment phase of a pathogen within the host tissues, the PAL activity often increases.

The increase in PAL activity indicates the activation of the phenylpropanoid pathway, and the plant defense enzymes activate the immune system. In several host-pathogen interactions, increased levels are correlated with incompatibility [57]. The product of PAL activity is trans-cinnamic acid, which is an immediate precursor for the biosynthesis of salicylic acid, a signal molecule in systemic acquired resistance [58](Klessig and Malamy, 1994).

ROS initiates a *plethora* of downstream signaling events essential for establishing defense mechanisms to prevent pathogen spread. Induction of plant systemic resistance by elicitors in several crops was associated with lignification and increased activities of defense gene products that are involved in the phenylpropanoid pathway and pathogenesis-related (PR) protein synthesis [59]. PR proteins are host-coded proteins induced by different types of pathogens and biotic stresses. Synthesis and accumulation of PR proteins have been well documented to play an essential role in plant defense mechanisms [60]. In the present study, the expression of *NPR1* and *PR1* genes were found to be up-regulated by Flg22_Xad1_ elicitor, while *PR2* was down-regulated or silenced (Fig. 6). Upon ROS burst, the altered redox state of the cell cause activation of NPR1 from an inactive oligomeric form to an active monomeric form in the cytoplasm, which will be translocated to the nucleus, where it activates the transcription of defense-related genes [9, 61]. Over-expression of the *NPR*1 gene suggests the activation of defense-related pathways in tobacco upon Flg22_Xad1_ treatment.

Moreover, NPR1 plays a crucial role in downstream of the salicylic acid-related defense signaling pathway [9]. This confirms the *Xad*1 flagellin-mediated systemic resistance in tobacco plants *via* salicylic acid signaling, and over-expression of the *PR1* gene revealed the primed state of the cells for defense response. Two critical events occur in defense priming; (i) accumulation of signaling or transcription factors, (ii) epigenetic changes that keep the plants in a “ready state” [61]. The epigenetic modification in plant immunity focuses on histone modifications by hormonal pathway-mediated defense signaling. *PR1* and *PR2* genes are well-defined markers to study chromatin modifications. In *Arabidopsis* and tobacco, over-expression of PR1 was related to increased histone acetylation in *PR1* locus [62–65]. While *PR2* negatively regulates immunity by affecting callose deposition upon pathogen challenge in *Arabidopsis* [66]. Down-regulation or silencing of the *PR2* gene upon Flg22 elicitation suggests the occurrence of active callose deposition at the site of infiltration to delineate a mechanical barrier and improved immune response. Accordingly, we confirm, that the recognition of *Xad*1 flagellin by FLS2 in tobacco plants activates a series of defense signaling pathways, *PR* genes or antimicrobial compounds, cell wall reinforcement, and PCD.

## Materials and methods

### Growth and protein production of Xad1 in M9 medium

The *X. axonopodis* pv. *dieffenbachiae* (*Xad*1) received from the Department of Plant Pathology, Tamil Nadu Agricultural University, Coimbatore was maintained in nutrient agar medium. The growth and extracellular protein secretion from *Xad*1 were monitored by culturing the cells in M9 Minimal broth supplemented with 0.2% glycerol as a sole carbon source. The flasks were maintained at 28 ºC under continuous shaking conditions at 200 rpm, and samples were drawn at regular intervals. The number of viable cells was determined by plating the cells in M9 media, followed by counting the colonies formed and later expressed in the Log of colony-forming units per milliliter (Log of CFU.mL^− 1^). The bacterial growth was recorded at an optical density of 600 nm; extracellular protein concentration was measured as described by Bardford method (1976) [67] and expressed in μg.mL^− 1^.

### Host-induced ***X. axonopodis*** pv. ***dieffenbachiae*** (Xad1) secretome

The plant extract was prepared by grinding 1 g of *Dieffenbachiae* leaves with 10 mL of phosphate buffer (pH 7) in sterile pestle and mortar. The extract was then filtered through Whatman No.1 filter paper followed by centrifugation at 10,000 rpm for 20 min. The supernatant was further clarified by filtering through a 0.2 μm membrane filter, and the plant extract was stored at − 20 ºC [22]. *X. axonopodis* pv. *dieffenbachiae* (*Xad*1) cells were cultured in M9 Minimal medium supplemented with 2% plant extract (*Dieffenbachiae* sp.) and maintained at 28 ºC under shaking conditions (200 rpm) until OD_600_ reached 0.6. The *Xad*1 cells cultured in M9 Minimal medium without plant extract served as the control. The secretome protein from the *Xad*1 culture was prepared by centrifugation at 10,000 rpm for 15 min at 4 ºC. The supernatant was filtered using a 0.2 μm membrane filter fitted to a filtration unit with vacuum suction. To concentrate the protein fractions, ammonium sulfate was then added to the supernatant to 60% saturation over 30 min with constant stirring and incubated overnight at 4 ºC. Later the pellet obtained by centrifugation at 13,000 rpm for 30 min at 4 ^o^C was resuspended in 500 μL phosphate buffer [68].

### Secretome analysis

#### Protein separation

The secretome protein concentration was estimated using the Bradford method [66]. The proteins were resolved on 12% SDS-PAGE using the Mini-PROTEAN Tetra cell vertical electrophoresis system (Bio-Rad) according to the method adopted by Laemmli (1970). An equal concentration (15 μg) of secretome protein from *Xad*1 with and without supplementation of plant extract was loaded onto the 12% SDS-PAGE gel and run at 30 mA for 3 h. Broad-range marker proteins (Precision Plus protein standard, Bio-Rad) containing 10–250 kDa molecular weight proteins served as the reference. The 2D gel electrophoresis was carried out as per the standard protocol mentioned (Supplementary Methods).

#### Biological activity of plant-defense-activating proteins from Xad1 secretome in tobacco

A single leaf from six-week-old tobacco plants was infiltrated with secretome (FliC_Xad1_) protein from *Xad*1 using a sterile 2mL syringe. Plants infiltrated with sterile water, buffer, and heat-killed secretome similarly served as control. The plants were maintained under controlled conditions for periodical observation and symptom development. The infiltered plants were periodically monitored for 48 h. The infiltrated leaves were removed and incubated with 0.1% DAB (3,3’-diaminobenzidine) solution under shaking conditions in the dark. A gentle vacuum was applied to the leaves using a desiccator for 3–5 min to absorb the stain. After 3–4 h, leaves were transferred to a bleaching solution and boiled for 15 min at 95 °C. Later the leaves were transferred to a fresh bleaching solution and allowed to stand for 30 min. The stained leaves were directly visualized and photographed. Three experimental replicates were maintained [69].

#### Enzyme extraction

Three-week-old tobacco plants were infiltrated with buffer (T_1_), FliC_Xad1_ (T_2_), heat-killed protein (T_3_), and plants without infiltration (T_4_) served as the control. Samples were collected from all the treatments at 0, 3, 6, 9, 12, 24, and 48 h after infiltration, immediately ground with liquid nitrogen, and stored at -80 ºC. One gram of powdered sample was extracted with 2 mL of sodium phosphate buffer, 0.1 M (pH 6.5) at 4 ºC, and centrifuged at 10,000 rpm for 20 min. The supernatant was used as an enzyme source for ROS enzyme assays.

#### Peroxidase assay

Peroxidase activity was assayed spectrophotometrically, according to Hartree (1955) [70]. The reaction mixture (1.5 mL of 0.05 M pyrogallol, 0.5 mL of enzyme extract, and 0.5 mL of 1% H_2_O_2_) was incubated at room temperature (28 ± 2 ºC). The change in absorbance at 420 nm was recorded at every 30-second interval for 3 min in a multimode microplate reader (Molecular Devices, USA). The boiled enzyme preparation served as the blank. The enzyme activity was expressed as a change in the absorbance of the reaction mixture (min^− 1^.g^− 1^) on a fresh weight basis [53].

#### Polyphenol oxidase assay

Polyphenol oxidase activity was determined by adopting the procedure of Mayer et al. (1966) [71]. The reaction mixture consists of 1.5 mL of 0.01 M sodium phosphate buffer (pH 6.5) and 200 μL of the enzyme extract. Catechol (200 μL; 0.01 M) was added to start the reaction, and the activity was expressed as a change in absorbance at 495 nm min^− 1^.g^− 1^ fresh weight of tissue.

#### Phenylalanine ammonia-lyase (PAL) assay

PAL activity was determined as the rate of conversion of L-phenylalanine to trans-cinnamic acid at 290 nm. Sample containing 0.4 mL of enzyme extract was incubated with 0.5 mL of 0.1 M borate buffer (pH 8.8) and 0.5 mL of 12 mM L-phenylalanine for 30 min at 30 ºC. The amount of trans-cinnamic acid synthesized was calculated using its extinction coefficient of 9630 M^− 1^.cm^− 1^ [72]. Enzyme activity was expressed as μmol trans-cinnamic acid min^− 1^.mg^− 1^ of fresh weight.

#### Native PAGE for polyphenol oxidase (PPO)

The enzyme was extracted by homogenizing 1 g of leaf tissue in 0.01 M potassium phosphate buffer (pH 7.0) followed by centrifugation at 20,000 g for 15 min at.

4 ºC was used as an enzyme source. After native electrophoresis, the gel was equilibrated for 30 min in 0.1% phenylene diamine containing 0.1 M potassium phosphate buffer (pH 7.0). The addition of 10 mM catechol, followed by gentle shaking, resulted in the appearance of dark brown discrete bands [73].

#### MALDI-TOF mass spectrometry (MS) and MASCOT analysis

The differentially expressed protein band from 1D SDS-PAGE gel was excised using a sterile scalpel and suspended in 5% acetic acid. The samples were packed in dry ice and shipped to Sandor Proteomics, Hyderabad, for MALDI-TOF analysis. The data were used to identify the proteins using the Mascot search tool (http://www.matrixscience.com). The following parameters were used for the database searches: Taxonomy: Proteobacteria, cleavage specificity, trypsin with 1 missed cleavage allowed, peptide tolerance of 50 ppm − 200 ppm for the fragment ions; with no modifications.

***In silico*analysis**.

The matching peptides were aligned using the BLAST (Basic Local Alignment Search Tool) program (www.ncbi.nlm.nih.gov/BLAST) with the query sequence as *Xanthomonas* sp. genome. The homology model of matching peptide, FliC, and Flg22 was performed using the automated Swiss-model server (https://swissmodel.expasy.org/) using PDB: 1UCU as a template. The docking analysis was performed using PyDockWeb server (https://life.bsc.es/pid/pydockweb) using PDB: 4MN8 as a receptor and Flg22 as a ligand, respectively [73, 74]. All the models were evaluated in the SAVES server (https://servicesn.mbi.ucla.edu/SAVES/). The structures were visualized using PyMol Molecular Graphics system version 0.97.

#### Peptide synthesis and its biological activity

Based on the molecular docking analyses, twenty-two amino acids at the N-terminal region of the flagellin protein obtained by MALDI-ToF analysis viz., Flg22 (QRLSTGSRINSAKDDAAGLQIA) were synthesized at Peptide Institute, Inc. (Osaka, Japan). The biological activity of the synthesized peptide was analyzed by the syringe infiltration technique. The flagellin peptide from *Xad*1 (0.5 μM, 1 μM, and 2 μM) was infiltrated into tobacco leaves using a sterile syringe, and the plants infiltrated with sterile water, BSA, and Flg22 peptide from *Pseudomonas syringae* (Gift of Dr.Vardis Ntoukakis, University of Warwick, UK) served as control. The hypersensitive response and hydrogen peroxide accumulation in the infiltrated leaves were analyzed, as described [75].

#### Differential expression of pathogenesis-related genes

Differential expression of the pathogenesis-related gene in response to FliC_Xad1_ protein was analyzed by semi-quantitative PCR. RNA was isolated from FliC_Xad1,_ water, and BSA-infiltrated leaf tissue using the NucleoSpin® RNA Plant kit (Macherey-Nagel, Germany). cDNA was synthesized from 1 μg of total RNA devoid of DNA contamination using High Capacity cDNA Reverse Transcription Kit (Applied Biosystems™, USA). RNA levels in the samples were normalized using *actin* gene. Pathogenesis-related genes viz., *PR1*, *PR2*, and *NPR1*were amplified using specific PR1 (F: 5’-CCCAAAATTCTCAACAAG-3’, R: 5’-TTAGTATGGACTTTCGCCTCT-3’); PR2 (F: 5’-ATGGCTTTCTTGCAGCTGCCCTTG-3’, R: 5’-GAGTCCAAAGTGTTTCTCTGTGATA-3’) and NPR1 (F: 5’-CTTTGGTCGTCCTCAAGCTC-3’, R: 5’-TCCATTGCAATTGTGCTTC-3’) primers respectively. Reverse Transcriptase PCR was performed in a final volume of 20 μLreaction mixture containing 1 μL of freshly synthesized cDNA, 1 μL of forward and reverse primer (10 μM) each, and 10 μL of PCR mix (Takara, USA). The following PCR program was used: 95 °C for 5 min, followed by 35 cycles of 95 °C for 50 s, 54 °C (*PR1*, *NPR1*, *Actin*)/ 56 °C (*PR2*) for 60 s and 72 °C for 60 s and a final extension of 72 °C for 10 min respectively.

## Conclusion

The study of plant-microbe interaction is essential to develop bioactive compounds like plant-defense-activating proteins, which could replace the use of chemical fungicide and pesticides, thereby maintaining the agro-ecosystem. Owing to the loss incurred by the plant-pathogenic genera *Xanthomonas*, the plant-pathogen interaction was studied in few species to identify the defense elicitors. *X. axonopodis* pv. *dieffenbachiae* is one of the *Xanthomonads* in which such studies were not reported. The present investigation focused on the identification and characterization of plant-defense-activating protein, flagellin identified from *X. axonopodis* pv. *dieffenbachiae* involved in host-pathogen interaction. Flagellin stimulated defense responses in tobacco plants and triggered the plant’s immune system prior to pathogen infection. The present study results can be exploited to invoke a defense response in other non-host plants.

### Electronic supplementary material

Below is the link to the electronic supplementary material.

Supplementary Material 1

## Data Availability

All data of this manuscript are included in the manuscript. No separate external data source is required. Any additional information required will be provided by communicating with the corresponding author via the official mail: usiva@tnau.ac.in.
