# Supplementary material for: Flagellin, a plant-defense-activating protein identified from Xanthomonas axonopodis pv. Dieffenbachiae invokes defense response in tobacco
Source: BMC Microbiol. 2023 Oct 5;23:284. doi: 10.1186/s12866-023-03028-z (PMC10552369; doi:10.1186/s12866-023-03028-z)
Supplement: Supplementary file 1 — Supplementary Material 1 [file 12866_2023_3028_MOESM1_ESM.doc]

**Supplementary Figure S1**

SDS-PAGE analysis of extracellular secreted protein using 1D SDS-PAGE (A), 2D SDS-PAGE (B), and MALDI-TOF MS analysis of the differentially expressed protein (C). In 1D SDS-PAGE, an additional band below 37 kDa (arrow pointed) was observed to be differentially expressed protein under plant extract induced conditions. Lane1 molecular mass marker; Lane 2 Uninduced *Xad*1; Lanes 3 induced *Xad*1; Lane 4 M9 broth with plant extract as a control. In 2D SDS-PAGE, the proteins with molecular mass from 20 to 29 kDa and p*I* value from 3.5 to 4.5 were identified. Peptide mass fingerprinting analysis was carried out with m/z values, and the peak file of *Xad*1 secretome indicated FliC or flagellin protein as a top matching hit with a score of 1319.94 with 9 unique peptide matches and a query coverage of 39.94.


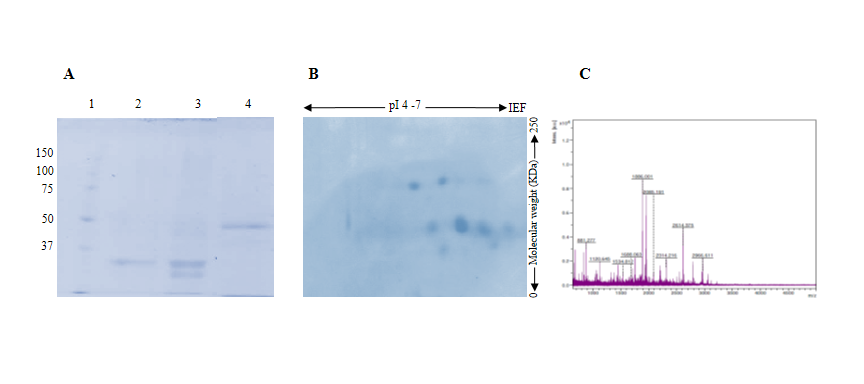


**Supplementary Figure S2**

Homology modelling of Flagellin (FliC_Xad1_) (A) and the Flg22_Xad1_ peptide (N terminal-ASMSTSIQRLSSGLRINSAKDDAAGLAISERFT-C-terminal) of the FliCFliC_Xad1_(B). The regions in Flg22_Xad1_spanning 22 amino acids (N terminal-QRLSSGLRINSAKDDAAGLAIS- C terminal) are critical for binding with FLS2 to initiate PAMP recognition and subsequent initiation of PTI.


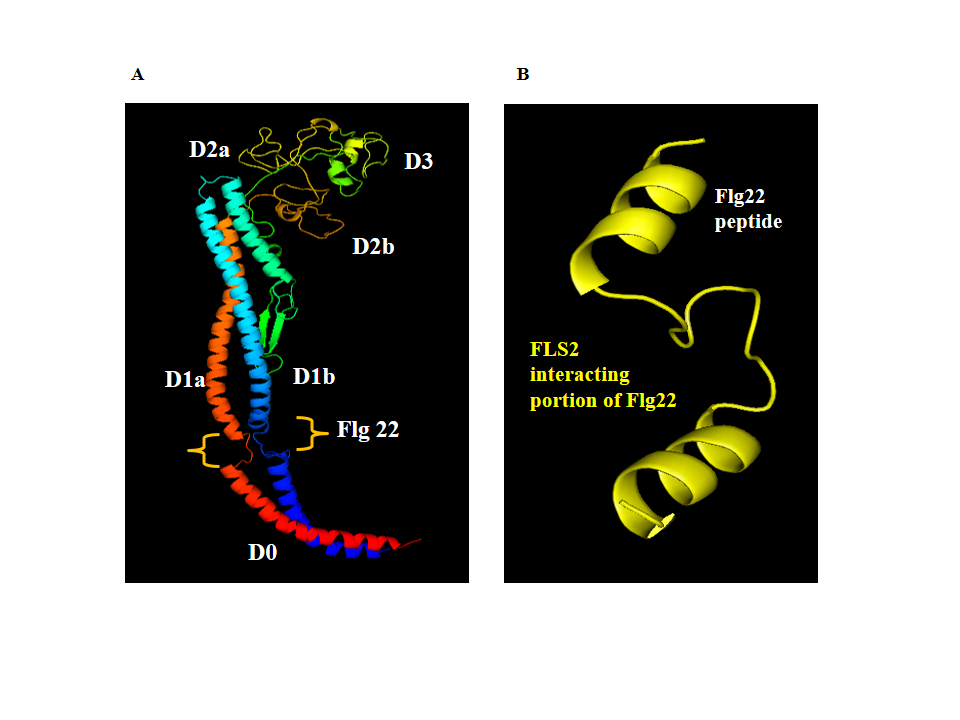


**Supplementary Figure S3**

Sequence homology of Xad1_Flg22_ peptide: Multiple sequence alignment showing sequence homology of the 22 amino acid epitope region (indicated in box) of Xad1_Flg22_ among important bacterial strains. The variability in the flg22 among different bacteria and strains can be seen which is responsible for invoking the immune response. The clustal consensus are indicated below the sequence alignment. The sequences used for the multiple sequence alignment with its respective accession number or sequence code (indicated in parenthesis) are *Xanthomonas axonopodis* (KPL49516), *X. axonopodis* Xac29-1 (AGH77465), *X. oryzaepv. oryzicola* (AEQ96507), *X. campestris pv. campestris* (ABC87263), *X. melonis* (WP_104588643), *X. citripv. glycines* (EWC52784), *X. cucurbitae* (WP_104603719), *A. tumefaciens* (WP_004432488), *E. amylovora* (CBX81556), *Rhizobium* sp. (WP_039845321), *R. solanacearum* (ACY25862), *R. etli* (WP_039620959), *R. leguminosarum* (WP_062945345), *Bradyrhizobium* sp. (GAJ33317), *Stenotrophomonas panacihumi* (WP_057648791), *Sphingomonas* sp. (3K8W), *Bacillus thuringiensis* (ABV23552).


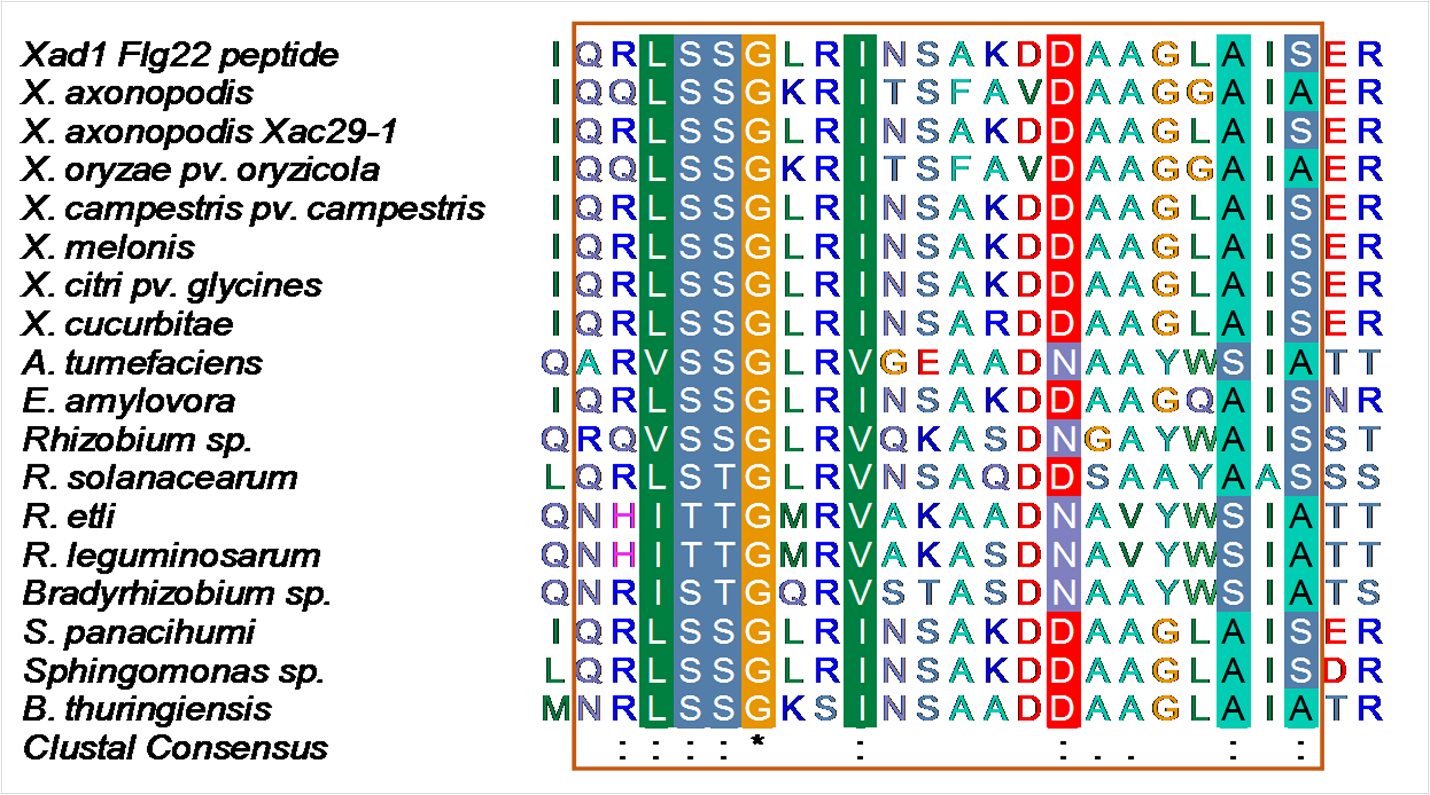


**Supplementary Method:**

**2-Dimensional electrophoresis (2DE)**

**Iso-electric focusing (IEF)**

For analytical gels, the 7 cm IPG (Immobiline pH Gradient) strips (pH 4.7 – 5.9) were rehydrated overnight with 125 μL of rehydration buffer (8M Urea, 2% CHAPS, Dithiothreitol [DTT] (7 mg per 2.5 mL of rehydration buffer) and 0.5% (v/v) IPG buffer pH 4.7 – 5.9) containing the required quantity of protein (14 μg) in a re-swelling tray (GE Healthcare., USA) at room temperature. The strips were rehydrated for about 12 - 14 h and subjected to the first dimension separation. IEF was carried out at 20 ºC in PROTEIAN i12 IEF Cell (BioRad). The samples were then focused at 50 V for 15 min, 150 V for 30 min, 150-250 V (linear) for 15 min, 4000 V (linear) for 5 h, 8000 V for 4 h, 8000 V for 40000 Vh and kept on hold at 250 V. The focused strips were equilibrated twice for 15 min in 2 mL equilibration solution. The first equilibration was performed in a solution containing 6M urea, 30% (w/v) glycerol, 2% (w/v) SDS, 1% (w/v) DTT and 50 mM Tris-HCl buffer. The second equilibration was performed in a solution modified by the replacement of DTT with 4% (w/v) iodoacetamide. Next, the IPG strips were then separated by 12% SDS-PAGE for the secondary dimensional analysis.

**Second-dimensional separation by SDS-PAGE**

The gel, glass plates, spacer and comb were cleaned thoroughly with water and then with 70% ethanol. The spacers were kept at the sides between the two glass plates. Separating gel was poured between the glass plates. Immediately after pouring, water was layered over the surface to minimize exposure of the polymerizing solution to oxygen so as to create a flat gel surface. The gel was allowed to polymerize for two hours. After the polymerization of the gel, water was poured off. Equilibrated IPG strips were rinsed with electrode buffer and placed on top of SDS gel. Standard protein marker (2 μL) was loaded near the gap between the strip and the gel plate edge. Then to place the strip in position, 2 mL of agarose sealing solution was overlaid. Gels were electrophoresed at constant current (25 mA) till the dye front reaches the bottom of the gel. Gels were removed from their gel cassettes and fixed in the fixative (4:1:5 of methanol: acetic acid: water) solution.
